# A pilot study exploring the efficacy and safety of herbal medicine on Korean obese women with metabolic syndrome risk factors: Double blinded, randomized, multicenter, placebo controlled study protocol clinical trial

**DOI:** 10.1097/MD.0000000000018955

**Published:** 2020-01-31

**Authors:** Hyun-ju Kim, Youme Ko, Hojun Kim, Yun-Yeop Cha, Bo-Hyoung Jang, Yun-Kyung Song, Seong-Gyu Ko

**Affiliations:** a Oriental Medicine Research Institute, College of Korean Medicine, Gachon University, Seong-nam-si; b Department of Korean Preventive Medicine, Graduate School; c Institute of Safety and Effectiveness Evaluation for Korean Medicine, Kyung Hee University; d Department of Korean Rehabilitation Medicine, College of Korean Medicine, Dongguk University, Seoul; e Department of Korean Rehabilitation Medicine, College of Korean Medicine, Sang-ji University, Wonju; f Department of Korean Rehabilitation Medicine, College of Korean Medicine, Gachon University, Seongnam-si, Republic of Korea.

**Keywords:** chowiseungcheng-tang, daesiho-tang, herbal medicines, metabolic syndrome risk factors, obesity, randomized controlled trial

## Abstract

**Background:**

Obesity is associated with metabolic syndrome, a condition that increases one's risk for heart disease and other conditions. The prevalence of obesity and associated diseases have steadily increased among Korean adults. The effect of the herbal medicines *Daesiho-tang* (DSHT) and Chowiseungcheng-tang (CST) on obesity have been reported. The purpose of this study is to evaluate the efficacy and safety of *Daesiho-tang* and Chowiseungcheng-tang on obese Korean women with high risk for metabolic syndrome.

**Methods/design:**

This study is a randomized, double-blinded, placebo-controlled, multi-center, 3-arm, parallel group clinical trial. A total of 120 participants will be enrolled and randomly assigned to the *Daesiho-tang* group, the Chowiseungcheng-tang group, or the placebo group in a 1:1:1 ratio using an internet-based randomization system at visit 2. Each group will be administered DSHT, CST, or placebo 3 times per day for 12 weeks. The primary outcome is to evaluate the changes in mean body weight of participants in the DSHT and CST groups and compare with those in the placebo group, and determine their statistical significance, if any, after 12 weeks. The secondary outcomes are the following: changes in body fat percentage and body fat mass, changes in waist circumference, waist-to-hip ratio, and body mass index, changes in serum lipids, fasting blood sugar, blood pressure, and C-reactive proteins (CRP) levels between visit 1 and visit 5 measurements. Changes in visceral fat volume determined through abdominal computed tomography, patient-reported health outcomes surveys—the Korean version of the Obesity-related Quality of Life and the Korean version of Eating Attitudes Test.

**Discussion:**

This study will provide research methodologies for evaluating the efficacy and safety of *Daesiho-tang* and Chowiseungcheng-tang on obese Korean women with high risk for metabolic syndrome.

Trial registration: ClinicalTrials.gov, NCT02651454. Registered on 11 January 2016.

Protocol version: The final approved version of the trial protocol is V1.3.(2017.11.10).

## Abstract 2

Supplemental Digital Content is available in the text

## 1 Introduction

The prevalence of obesity and related diseases has steadily increased among Korean adults. Obesity is associated with increasing risks for type 2 diabetes mellitus (T2DM), hypertension (HTN), metabolic syndrome, and other dyslipidemia diseases. According to previous studies, the prevalence of obesity increased from 29.7% to 32.4% from 2009 to 2015, with an estimated cost of $ 1.8 billion as per for available 2015 data.^[1–3]^

Various therapeutic strategies using conventional medications to treat or prevent obesity have been proposed, including many of their side effects which may limit their use.^[4]^ Complementary and alternative medicine (CAM) may offer some promise here as the beneficial effects of herbal medicine on obesity have been reported.^[5]^

Therefore, research is needed not only to confirm effective in obesity but also, the safety of use in human clinical studies. *Daesiho-tang* (DSHT) is a herbal formulation in traditional Korean medicine; it has been registered and authorized for use in Korea by the Korea Food & Drug Administration. According to the *Donguibogam*, it is used for the treatment of acute febrile illness, acute pneumonia, habitual constipation, and chronic gastritis.^[6]^ The anti-obesity effect of DSHT has been shown in preclinical studies. Obesity-induced mice in the DSHT group showed greater reductions in the body weight, and liver and fat tissue mass when compared with the control group. In hematological experiments, total cholesterol (TC), triglyceride (TG), glucose tolerance, and HDL cholesterol were significantly reduced in high-fat diet-fed mice. Quantitative analysis of denaturing gradient gel electrophoresis (DGGE) showed that bacterial gene expression in HFD mice was significantly increased in the DSHT treated group. As a result of measuring liver tissue using RT2 PCR, it was observed that several genes in the DSHT treatment group regulated cholesterol metabolism by expressing them up and down.^[7]^

*Chowisengcheong-tang* is an anti-obesity herbal medicine for *Taeeumin* made by *Dongeuisusaebowon*.^[8]^ It is currently marketed under the approval of KFDA for the treatment of abdominal bloating and distention after meals, and leg muscle weakness. According to findings of previous human subject studies, significant weight loss was achieved after receiving combined treatment with electro acupuncture and CST.^[9]^ However, the effect of CST alone on obesity has not yet been confirmed in clinical studies.

There is either a lack of or inadequate reporting of the scientific evidence of the effects of DSHT and CST on obesity in clinical studies. This clinical trial is intended to help fill this gap by evaluating the effectiveness and safety of the 2 herbal medicines DSHT and CST in obesity. We designed a randomized, placebo-controlled, multi-center, clinical trial to evaluate the efficacy and safety of the pharmacological intervention, DSHT and CST on obese women with high risk for metabolic syndrome.

## 2 Methods/design

### 2.1 Study design

This trial is a randomized, double-blinded, three-arm, placebo-controlled, multi-center, study that will be conducted at the following sites: Gachon University Gil Korean Medical Hospital, Dongguk University Ilsan Oriental Hospital, and Sangji University Oriental Medical Center. The total study duration is approximately 13 weeks from the screening visit. A schedule of this study flow is shown in Table 1.

**Table 1:**
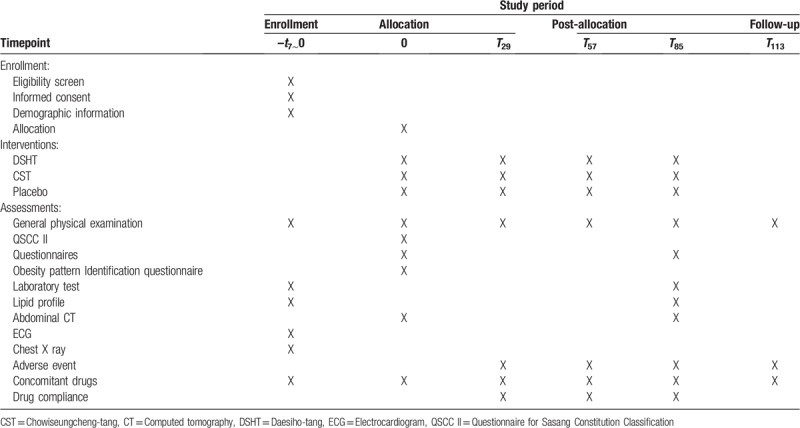
Study flow of the trial.

## 3 Study objectives

The main objective is to evaluate the efficacy and safety of DSHT and CST on obese Korean women with high risk for metabolic syndrome.

### 3.1 Recruitment

The study participants will be recruited through advertisement for human research study which includes the following information: the purpose of the study, study duration, eligibility criteria, and the detailed information on the investigational drugs. The advertisement notice, posted on bulletin boards inside and outside the hospitals, will invite potential study participants to voluntarily contact the site trial staff for eligibility assessment.

### 3.2 Participants

#### 3.2.1 Inclusion criteria

Criteria for inclusion of participants are as follows:

1. Females aged 18 to 65 years.
2. Patients who have any of the following conditions:
  1) BMI 30 kg/m^2^ or over;
  2) BMI 25 to 29.9 kg/m^2^, who have at least one of the following risk factors;
    - Patients who have been diagnosed with hypertension and currently under adequate treatment with antihypertensives, or individuals with systolic blood pressure (SBP) ≥140 mmHg and diastolic blood pressure (DBP) ≥90 mmHg at the screening visit.
    - Patients who have been diagnosed with T2DM and are currently on adequate treatment or individuals with fasting blood glucose >126 mg/dL at the screening visit.
    - Patients who have been diagnosed with hyperlipidemia and are currently under adequate treatment, or individuals with total cholesterol ≥200 mg/dL or triglycerides ≥150 mg/dL at screening visit.
3. Subjects who have agreed to a low-calorie diet during the trial.
4. Patients who have signed the written informed consent form.

### 3.3 Exclusion criteria

Criteria for exclusion of participants are as follows:

1. Patients with endocrine diseases such as hypothyroidism, Cushing syndrome, etc, which can affect the weight readings.
2. Patients with cardiovascular diseases, such as, heart failure, angina pectoris, myocardial infarction, and stroke.
3. Patients with uncontrolled hypertension with SBP >145 mmHg or DBP >95 mmHg.
4. Patients with severe renal or liver impairment (serum creatinine [SCr] > 2.0 mg/dL or 2.5 times higher than the maximum values of alanine transaminase [ALT], aspartate aminotransferase [AST], and alkaline phosphatase [ALP] in patients with normal renal and liver function.).
5. Patients with a surgical history for weight reduction such as bariatric surgery.
6. Patients with history of or current eating disorder such as anorexia nervosa or bulimia nervosa.
7. Patients who have experienced a 10% weight reduction within the past 6 months.
8. Those who decided to stop smoking within the last 3 months or who have an irregular smoking habit.
9. Patients with cholelithiasis.
10. Patients with narrow angle glaucoma.
11. Patients receiving treatment with controlled substances including antidepressants, antiserotonins, barbiturates, antipsychotics, Central nervous system stimulants.
12. Patients on any medications that may have an effect on absorption, metabolism, and excretion of drugs and weight within the last 3 months; these include appetite suppressants, laxatives, oral steroids, thyroid hormone, amphetamine, cyproheptadine, and phenothiazine.
13. Patients who are in the childbearing age and do not agree to use effective contraceptive methods during the study period.
14. Patients who have participated in any clinical trial within the past month.
15. Patients judged unable to follow instructions of the trial by clinical trial investigators.

### 3.4 Subject withdrawal criteria

The subject withdrawal criteria are as follows: detection of eligibility violations; occurrence of a serious adverse event; subject has an acute reaction (such as allergy or shock) due to administration of the investigational product; detection of a systemic disease that was not discovered at the screening; <70% adherence rate; subject's withdrawal of consent; use of any forbidden medication or treatment during the trial that could affect the study result; uncooperative subject; occurrence of other significant protocol violations; subject who cannot follow up; subject unable to progress because of worsening of pre-existing disease; investigator's decision to terminate the process for the sake of the subject's health. The participants who are withdrawn after randomization will be followed up to examine outcomes.

### 3.5 Sample size

This trial is a pilot study to examine the feasibility of a full randomized controlled trial for evaluating the efficacy and safety of DSHT and CST in obesity and to determine the effect size for further large-scale studies. A sample cohort comprising of 120 patients have been considered after assessing their frequency of clinic visits, availability of study staff, funding availability, and dropout rates at each participating site.

### 3.6 Randomization and blinding

The Institute of Safety, Efficacy, and Effectiveness Evaluation for Korean Medicine (ISEE), the Contract Research Organization (CRO), will generate random sequences using the R program. Randomization number assignment via the web-based system will be performed at visit 2 by the clinical research coordinator (CRC) or research assistants at each site. The subjects in this trial will be assigned to one of three groups with an allocation ratio of 1:1:1. All participants, investigators, and monitors will be blinded. No one, with the exception of the statisticians at ISEE, will be aware of which random numbers refer to either of the treatment groups or the placebo group. The randomization table kept in the opaque sealed envelope by the CRO will be opened according to Standard Operating Procedures (SOPs).

### 3.7 Study procedures

After enrollment, participants will be receiving the assigned Investigational product (IP) and will be prohibited from taking any drugs which can affect weight loss for 12 weeks. The IP will be orally administered as 6 g of the given granules to be taken with water 3 times a day after meals. The daily dosage of both IPs is based on the Ministry of Food and Drug Safety (MFDS) guidelines. The study participants will be asked to return any remaining drugs at 4 weekly intervals to assist in the determination of adherence.

### 3.8 Interventions

In this trial, we plan to use DSHT from Hanpoong Pharm & Foods Co., Ltd., and CST from Hanjoong Pharmaceutical company. The placebo production, IP repackage, and IP distribution process were performed by Hanpoong Pharm & Foods Co., Ltd.

DSHT consists of 8 medicinal herbs: Paeoniae radix (1.00 g), Scutellariae radix (1.00 g), Bupleuri radix (2.0 g), Pinelliae rhizome (1.33 g), Zingiberis rhizome (1.67 g), Rhei undulati rhizome (0.67 g), Zizyphus fructus (1.00 g), and Ponciri fructus (0.67 g).

CST consists of 12 medicinal herbs: Coicis Semen (3.75 g), Castanea mollissima (3.75 g), Raphani Semen (1.875 g), Epedrae Herba (1.25 g), Platycodi Radix (1.25 g), Liliopis Tuber (1.25 g), Schizandrae Fructus (1.25 g), Acori Rhizoma (1.25 g), Polygalae Radix (1.25 g), Asparagi Radix (1.25 g), Zizyphi Semen (1.25 g), Longanae Arillus (1.25 g).

Each raw material will be manufactured in Hanpoong and Hanjoong, respectively, and both will be collected and packaged as IP by Hanpoong Pharm & Foods Co., Ltd.

The placebo product consists of lactose, corn starch, and food colorings, and possesses an appearance, shape, weight, taste, and color similar to DSHT and CST. The treatment drug and placebo will be supplied by Hanpoong Pharm & Foods Co., Ltd.

### 3.9 Primary outcome measurement

The primary outcome is to evaluate the changes in mean body weight in the DSHT and CST groups compared with the placebo group and determine their statistical significance, if any, after 12 weeks.

### 3.10 Secondary outcome measurements

The secondary outcomes will focus on changes of following obesity related measurements between baseline and post treatment: mean weight reduction, body mass index (BMI), body fat, waist and hip circumferences, lipid profile, C-Reactive protein (CRP), volume of visceral fat, Korean version of Obesity-related Quality of Life (KOQoL), The Korean Version of Eating Attitudes Test-26 (KEAT-26).

KOQoL is a validated outpatient-based quality of life assessment questionnaire on obese patients. It consists of 6 areas (psychosocial health, physical health, work and family affairs, daily life, sexual relationships, and relationship with food) with a total of 15 items. Each item is a 4-point Likert Scale ranging from “absolutely not” (1 point) to “always” (4 points). The total maximum score is 60 points. Higher score represents low quality of life.^[10]^ KEAT-26 is a validated Korean-translated version of EAT-26. It is 6-level Likert scale which consists of 11 items concerning anorexia, 7 items concerning binging or overeating, and 8 items concerning control of eating behavior. Higher score represents more problematic mealtime behavior.^[11]^

### 3.11 Safety outcomes

All variables related to the safety assessment of the study such as vital signs, physical examination, and various laboratory test results, including hematologic test, biochemical test, and urine test, as well as adverse events will be documented on the case report form (CRF) at every visit.

### 3.12 Other instruments

Questionnaire for Sasang Constitution Classification II (QSCC II) and Korean Obesity Pattern Identification Questionnaire (KOPIQ) are the validated Korean medicine based typologies tools which helps to evaluate the efficacy and safety on different subgroups. QSCC II is a validated survey tool that diagnose into the 4 constitutional types based on the Korean traditional constitutional medicine: Taeyangin, Soeumin, Taeumin, and Soyangin. It consists of 121 items, comprising of psychological (54 items), behavioral (36 items), and physical characteristics (11 items), as well as health and disease questions (20 items).^[12]^ The result of QSCC II is automatically calculated by Win QSCC II version software (SordMedicom & sord OMS, Seoul, Korea). KOPIQ is a validated pattern identifications questionnaire on obesity which was developed by the Korean Institute of Korean Medicine (KIOM). It consists of 52 items with 4 domains: general symptoms, emotional, digestive, and vitality. The final pattern is classified by KOPIQ scoring algorithm from KIOM.^[13]^

### 3.13 Statistical analysis

#### 3.13.1 Efficacy assessment

The demographic data at the baseline will be analyzed using a *t* test for continuous data and a chi-square test for categorical data. Analysis of the primary endpoint will be performed using intention-to-treat (ITT) datasets. Per-protocol (PP) datasets will also be analyzed as a reference. The term ITT dataset refers to a set of data regarding all randomly assigned participants. Missing data will be assigned using the last-observation-carried-forward (LOCF) analysis method. PP datasets include data on only the participants who completed the trial with no protocol violations or deviations.

The secondary endpoint data will be analyzed using both ITT and PP datasets. The average weight change before and after treatment will be analyzed using a paired *t* test for intragroup data and a Student *t* test for intergroup data. The average weight during the treatment phase will be analyzed using repeated measures analysis of variance (ANOVA) tests. Other secondary data will be analyzed identically to the average weight change, with the exception of glucose measurements and questionnaire results, which will be compared only before and after treatment between the groups. We also use ANOVA tests to analyze subgroups of QSCC II and OPIQ. The present study has no plan for an interim analysis.

### 3.14 Safety assessment

The participants are instructed to report any adverse events which may occur while taking the interventions. All possible adverse events will be described in the CRF. The investigator will withdraw any participant experiencing a serious adverse event. This will be reported to the MFDS within 15 working days of withdrawal according to the Korea Good Clinical Practice (KGCP) guidelines. Participants who have withdrawn will receive appropriate treatment. The investigator will have full responsibility for the safety of participants. For safety assessments, a liver function test, blood cell count test, physical examination, and urine analysis will be conducted at baseline and at visit 5. Any loss caused by the trial will be reimbursed via insurance. The safety datasets will include the participants who are administered DSHT, CST, or placebo at least once. The incidences of physical examination and self-reported adverse events will be compared using a chi-square test. A liver function test, blood cell count test, and urine analysis will be compared between the groups. The data will be categorized into either normal or abnormal groups according to their respective normal ranges. Differences between the groups will be assessed via a chi-square test.

### 3.15 Data and safety monitoring

The ISEE will monitor the clinical trial. The monitoring will begin once the first participants complete the required number of visits. All investigational institutions will be monitored based on the SOPs while this trial is in process. Auditing is not scheduled for the present study. To improve data quality, range checks for data values and double data entry will be conducted. Any other committee such as a coordinating center, steering committee, or endpoint adjudication committee will not be applicable to the present study.

### 3.16 Ethics and dissemination

This trial is conducted to comply with the Declaration of Helsinki 2008 and/or the regulations of the GCP principles in the Korean MFDS. This trial has received the approval of the institutional review boards (IRBs) of all three institutions: Gachon University Gil Korean Medical Hospital (15–102), Dongguk University Ilsan Oriental Hospital (2015–07), and Sangji University Oriental Medical Center (SJ IRB 120607). The current protocol is version 1.5, and it is developed according to the SPIRIT checklist (see Additional file 1). When important protocol changes occur, the changes must be approved by the IRBs before implementation. All items have been drawn from the World Health Organization (WHO) Trial Registration Data Set. Written informed consent will be obtained prior to the study by the investigator (see Additional file 2). Moreover, we have contracted liability insurance for patient safety. The confidentiality of personal information will be ensured. Each participant will be assigned a trial identification number at enrolment. For the duration of the entire trial, data will be handled based on the trial identification number. All records will remain secure in a locked cabinet or password-protected computer files, both for the duration of the trial and after the trial has concluded. Only investigators will retain the right to access the data. The results of the study will be disseminated through scientific journals or scientific conference presentations. So far, no public access to the full protocol, participant-level datasets, or statistical code is planned.

## 4 Discussion

This multi-center, randomized, 3-arm placebo-controlled study is to investigate the efficacy and safety of DSHT and CST in female obese patients with high risk for metabolic syndrome. Findings from preclinical experiments have demonstrated the anti-obesity effects of DSHT in the presence of high glucose levels^[7]^ and CST has proved effective in increasing HDL levels. However, no previous clinical trials have reported their effects in obese patients with concomitant obesity-related diseases. Traditional herbal medicines are used widely in clinical practice for weight management in East Asia including Korea, but their use is contentious owing to the lack of high-quality scientifically-based evidence.^[14]^ Building on our study with evidence-based obesity treatment strategy for obese female patients with high risk for metabolic syndrome, this study may be the first to examine the efficacy and safety of DSHT and CST on in obese patients with high risk for metabolic syndrome.

There will be several limitations in this study. First of all, although we will obtain participants’ agreement on low calorie diet during the study period, daily food intake will not be fully controlled. Thus, participants will be encouraged to check their daily calorie intake before the clinic visits through continuous patient–researcher communication. Second, a short-term drug administration period has been applied, with small number of participants; thus, a larger, long-term trial is warranted to understand the impact on long-term weight and fat management.

In conclusion, this pilot study will provide support for the design and methods and determining the feasibility of the larger clinical trial among obese female patients in Korea. If the trial medicines are proven safe and efficacious, this study's findings will be used as an evidence for the use of DSHT and CST in female obesity treatment in Korean medicine clinics and hospitals.

## Author contributions

**Conceptualization:** Hojun Kim, Yun-Yeop Cha, Bo-Hyoung Jang, Yun-Kyung Song, Seong-Gyu Ko.

**Methodology:** Hojun Kim, Yun-Yeop Cha, Bo-Hyoung Jang, Yun-Kyung Song, Seong-Gyu Ko.

**Project administration:** Yun-Kyung Song, Seong-Gyu Ko.

**Statistical consultation:** Bo-Hyoung Jang.

**Supervision:** Yun-Kyung Song, Seong-Gyu Ko.

**Writing – original draft:** Hyun-ju Kim, Youme Ko.

**Writing – review & editing:** Hojun Kim, Yun-Yeop Cha, Bo-Hyoung Jang, Yun-Kyung Song, Seong-Gyu Ko.

Hyun-ju Kim orcid: 0000-0002-9362-3041

Youme Ko orcid: 0000-0002-3458-6738

Hojun Kim orcid: 0000-0003-1038-0142

Yun-Yeop Cha orcid: 0000-0002-2360-622X

Bo-Hyoung Jang orcid: 0000-0002-2141-3483

Yun-Kyung Song orcid: 0000-0003-4478-4030.

Seong-Gyu Ko orcid: 0000-0001-9260-8889

## Supplementary Material

Supplemental Digital Content

## References

[1] Seo MH Kim YH Han K Prevalence of obesity and incidence of obesity-related comorbidities in Koreans based on National Health Insurance Service Health Checkup Data 2006-2015. J Obes Metab Syndr 2018;27:46–52.31089540

[2] Park JH Yoon SJ Lee H Burden of disease attributable to obesity and overweight in Korea. Int J Obes (Lond) 2006;30:1661–9. 16534516 10.1038/sj.ijo.0803321

[3] Kang JH Jeong BG Cho YG Socioeconomic costs of overweight and obesity in Korean adults. J Korean Med Sci 2011;26:1533–40. 22147988 10.3346/jkms.2011.26.12.1533 PMC3230011

[4] Yi DY Kim SC Lee JH Clinical Practice Guideline for the Diagnosis and Treatment of Pediatric Obesity: Recommendations from the Committee on Pediatric Obesity of the Korean Society of Pediatric Gastroenterology Hepatology and Nutrition. Pediatr Gastroenterol Hepatol Nutr 2019;22:1–27. 30671370 10.5223/pghn.2019.22.1.1 PMC6333581

[5] Liu Y Sun MY Yao H Herbal medicine for the treatment of obesity: an overview of scientific evidence from 2007 to 2017. Evid Based Complement Alternat Med 2017;2017:8943059 doi: 10.1155/2017/8943059. Epub 2017 Sep 25. 29234439 10.1155/2017/8943059 PMC5632873

[6] Heo J Donguibogam. Seoul: Namsandang; 1980. pp. 1, 69, 82, 104, 597, 601. Korean.

[7] Hussain A Yadav MK Kim HJ Daesiho-Tang is an effective herbal formulation in attenuation of obesity in mice through alteration of gene expression and modulation of intestinal microbiota. PLoS One 2016;11:e0165483 doi: 10.1371/journal.pone.0165483. eCollection 2016. 27812119 10.1371/journal.pone.0165483 PMC5094769

[8] Lee J-M Dongeuisusebowon-Sasang-Chobonkwon, Translate by S. G. Park. Seoul, Korea: Jiwentang; 2003.

[9] Seo DM Lee S H Lee F D Clinical observation on effects and adverse effects of choweseuncheng-tang on obesity patients. J Korean Acupunct Moxibustion Soc 2005;22:145–53.

[10] Park HS Development of Korean version of obesity-related quality of life scale. Kor J Obes 2003;12:280–93.

[11] Rhee MK A standardization study of the Korean version of eating attitude test26 I: reliability and factor analysis. Korean J Psychosomatic Med 1998;6:155–75.

[12] Choi S A study on the association between Sasang Constitutions (QSCC II) and Huh's morphological diagramming. Korean J Oriental Med 2002;8:75–92.

[13] Kang BG Moon JS Choi SM A reliability analysis of syndrome differentiation questionnaire for obesity. Korean J Orient Med 2007;13:109–14.

[14] Korean Institute of Oriental Medicine (KIOM, the society of Korean medicine for obesity, research). Korean Medicine Clinical Practice Guideline for Obesity. Daejeon, South Korea: Elsevier; 2016.

