# Supplementary material for: A pilot study exploring the efficacy and safety of herbal medicine on Korean obese women with metabolic syndrome risk factors: Double blinded, randomized, multicenter, placebo controlled study protocol clinical trial
Source: Medicine (Baltimore). 2020 Jan 31;99(5):e18955. doi: 10.1097/MD.0000000000018955 (PMC7004663; doi:10.1097/MD.0000000000018955)
Supplement: Supplemental Digital Content [file medi-99-e18955-s001.pdf]

[Appendix] Informed Consent Form (ver 1.1)

A Pilot Study Exploring the Efficacy and Safety of Herbal Medicine on Korean Obese Women with Metabolic Syndrome Risk Factors - Double Blinded, Randomized, Multicenter, Placebo Controlled Clinical Trial

□ I have read the participant information sheet and I understood the purpose, methods, expected effect, possible risk, and information management collected in the study with a full explanation.

□ I have had the opportunity to ask questions about it and any questions that I have asked have been answered to my satisfaction.

□ I was also informed that I can withdraw the agreement and receive appropriate treatment if any adverse event occurs.

□ I understood the explanation about collecting, using and providing personal information.

□ I have a copy of this consent form and information sheet.

□ I have been given sufficient time to consider and I consent voluntarily to participate as a participant in this research.

Participant

Print Name ___________________ Signature ___________________ Date ___________________

Legal representative (if necessary) (Relationship:)

Print Name ___________________ Signature ___________________ Date ___________________

Witness (if necessary)

Print Name ___________________ Signature ___________________ Date ___________________

Researcher/person taking the consent

Print Name ___________________ Signature ___________________ Date ___________________
